# Toward Wearables for Bruxism Detection: Voluntary Oral Behaviors Sound Recorded Across the Head Depend on Transducer Placement

**DOI:** 10.1002/cre2.70001

**Published:** 2024-09-22

**Authors:** Mohammad Khair Nahhas, Jens Christoph Türp, Philippe Cattin, Nicolas Gerig, Elisabeth Wilhelm, Georg Rauter

**Affiliations:** ^1^ BIROMED‐Lab, Department of Biomedical Engineering University of Basel Allschwil Switzerland; ^2^ Division of Temporomandibular Disorders and Orofacial Pain, Department of Oral Health and Medicine University Center for Dental Medicine Basel UZB Basel Switzerland; ^3^ CIAN, Department of Biomedical Engineering University of Basel Allschwil Switzerland; ^4^ Discrete Technology and Production Automation, Engineering and Technology Institute Groningen, Faculty of Science and Engineering University of Groningen Groningen The Netherlands

**Keywords:** jaw clenching, sound, teeth grinding

## Abstract

**Objectives:**

Bruxism is a parafunctional orofacial behavior. For diagnosis, wearable devices that use sounds as biomarkers can be applied to provide the necessary information. Human beings emit various verbal and nonverbal sounds, making it challenging to identify bruxism‐induced sounds. We wanted to investigate whether the acoustic emissions of different oral behaviors have distinctive characteristics and if the placement of the transducer has an impact on recording the sound signals.

**Material and Methods:**

Sounds from five oral behaviors were investigated: jaw clenching, teeth grinding, reading, eating, and drinking. Eight transducers were used; six were attached to the temporal, frontal, and zygomatic bones with the aid of medical tape, and two were integrated into two commercial earphones. The data from 15 participants were analyzed using time‐domain energy, spectral flux, and zero crossing rate (ZCR).

**Results:**

In summary, all oral behaviors showed distinct characteristic features except jaw clenching, though there was a peak in the recording, possibly due to tooth tapping, before its expected onset. For teeth grinding, the transducer placement did not have a significant impact (*p* > 0.05) based on energy, spectral flux, and ZCR. For jaw clenching, the transducer placement had an impact with regard to spectral flux (*p* < 0.01). For reading and eating, the transducer placement had a significant impact with regard to energy (*p* < 0.05 for reading, *p* < 0.01 for eating), spectral flux (*p* < 0.001 for reading, *p* < 0.01 for eating), and ZCR (*p* < 0.001 for both reading and eating). For drinking, the transducer placement only had a significant impact with regard to ZCR (*p* < 0.01).

**Conclusions:**

We were able to record the sounds of various oral behaviors from different locations on the head. However, the ears were an advantageous location to place the transducer, since they could compensate for various head movements and ear devices are socially tolerable.

## 1 Introduction

Bruxism, a parafunctional orofacial behavior, is characterized by teeth grinding or jaw clenching that can happen during sleep or wakefulness (Lobbezoo et al. 2018). Approximately 8% of the population suffers from severe sleep bruxism, which requires therapy (Castroflorio et al. 2013; Lavigne and Montplaisir 1994). Bruxism is related to multiple health risks such as emotional stress, drug use, or the use of certain medications (Kuhn and Türp 2018). It may lead to many health problems, such as temporomandibular pain, tooth wear, or anterior‐disc displacement (Ramírez, Sandoval, and Ballesteros 2005; Kim 2016). Polysomnography (PSG) with audio and video recordings is the gold standard for diagnosing bruxism (Svensson et al. 2017). PSG is resource‐intensive and requires an overnight stay in a sleep laboratory. Self‐reports are used to indicate the presence of sleep bruxism. However, a shortcoming of self‐report is that it does not allow for the determination of the severity of sleep bruxism (Raphael et al. 2015). Consequently, tiny dedicated sensors to monitor bruxism are necessary, not only to monitor sleep bruxism and its severity but also to monitor awake bruxism as well (Manfredini et al. 2020).

Developments in wearable devices have allowed health monitoring in real‐world settings for longer durations (Seshadri et al. 2019). The possibility of using such wearable devices for monitoring bruxism at home could be a cost‐effective alternative to PSG (Castroflorio et al. 2013; Manfredini et al. 2020; Deregibus et al. 2014; Prasad et al. 2019). For instance, monitoring sleep bruxism was investigated in real‐world settings using a portable device, Bruxoff (Spes Medica, Battipaglia, Italy). Bruxoff consists of an electromyography (EMG) system that monitors the activity of the masseter muscles and an electrocardiography system that monitors heart rate (Castroflorio et al. 2013; Deregibus et al. 2014). In 2019, a wireless EMG sensor was developed for monitoring the activity of the masseter muscles and for classifying different oral tasks such as smiling or chewing gum. Considering the small sample of healthy participants, the research group concluded that their device has the potential to be used for monitoring the activity of masticatory muscles (Prasad et al. 2019). Bruxoff targets sleep bruxism but does not monitor awake bruxism. In addition, potential users may not tolerate wearing electrodes on their cheeks during the day, and the device is sensitive to activities unrelated to bruxism. Consequently, we found that the current devices are limited in their scope. They focus on either sleep or awake bruxism, and there is a lack of real‐world testing where activities unrelated to bruxism are present.

Acoustic emissions are used in wearable devices to record or monitor oral behaviors, such as eating (Amft et al. 2005; Päßler and Fischer 2011) or talking (McBride et al. 2011; Lezzoum, Gagnon, and Voix 2014). They are also used as biomarkers for detecting health problems such as knee osteoarthritis (Schlüter et al. 2019) or irritable bowel syndrome (Du et al. 2019). In addition, monitoring heart and breathing rates from the ear has been investigated using an earpiece equipped with two microphones, one placed inside the ear canal and another that picks up sounds from the environment (Martin and Voix 2017). The possibility of monitoring eye movements from the ear has been investigated using in‐ear microphones as well. One such study observed that when the eyes moved, the eardrums moved, which changed the pressure in the occluded ear (Gruters et al. 2018). Tongue movements have also been detected from the ear using an in‐ear barometer for hands‐free interaction (Maag et al. 2017). Most importantly, for the present context, acoustic emissions of tooth contact—extracted from the ear or other locations—have been used in dentistry to assess occlusion properties. In the second half of the 20th century, there were attempts, referred to as “gnathosonics,” to use stethoscopes to detect the acoustic emissions generated by the temporomandibular joints or the occlusion of teeth during jaw movements (Watt 1966). Later, instead of recording the sounds from the zygoma, as described in Watt (1966) and Prinz (2000) tooth‐contact sounds were recorded from the ear with transducers built into a portable audio player or over‐ear device. In 2016, tooth‐contact sounds were recorded using bone conduction microphones attached to the temporal bone to realize a hands‐free user interface (Ashbrook et al. 2016). From these investigations, we inferred that acoustic emissions could be used to detect bruxism‐induced events. During teeth grinding, sounds are transmitted through bone propagation in the head, whereas during jaw clenching, detectable signals can be produced via two pathways: (a) the activation of middle‐ear muscles can alter the pressure in an occluded ear (Slegel et al. 1991; Ramírez, Ballesteros, and Pablo Sandoval 2007) or (b) vibrations caused by the activity of mastication muscles can propagate in the vicinity of the muscles (Petersen and Christensen 1973; Tortopidis, Lyons, and Baxendale 1998; Nakamura et al. 2005). We confirmed the likelihood of this hypothesis in a previous case study (Nahhas et al. 2022). In addition, the possibility of detecting teeth‐grinding sounds and other nonverbal orally induced sounds has been investigated for telecommunication (Bouserhal et al. 2018; Chabot et al. 2021) in studies following up on the work of Martin and Voix (2017). The latter investigation successfully classified various verbal and nonoral sounds using an earplug in a controlled environment. Another study recorded mandibular movement in addition to acoustic emissions to detect bruxism (Alfieri et al. 2021). The transducers they used were two three‐axis inertial measurement units (IMUs) attached to the chin and the masseter muscle and a microphone attached to the cheek to record the sounds.

It is important to note that bruxism is a parafunctional behavior that may occur throughout the day, so the location and the type of the detection device should be ergonomically and aesthetically tolerable. Several factors influence the quality of the recording, such as the accurate placement of the transducer, the relative strength of the signal when comparing different transducer placements, and the comfort of wearing the transducer for an extended period of time. One of the relevant questions to be answered is as follows: if bruxism‐induced acoustic emissions can be recorded from the head, which location on the head is most sensitive to the differences in the sounds created by different behaviors?

In this work, we aimed to investigate whether the acoustic emissions of different oral behaviors have distinctive characteristics and whether transducer placement has an impact on recording the sound signals.

## 2 Methods

### 2.1 Setup

**Figure 1:**
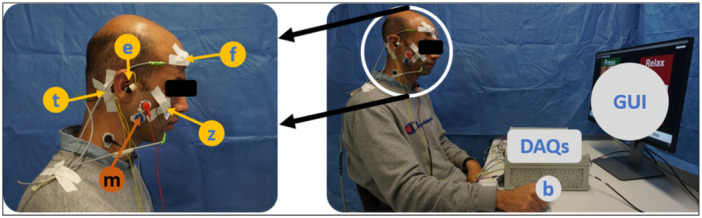
Experimental setup. The left picture shows how eight transducers were distributed symmetrically between the left side and right side of the head: (f) frontal bone, (z) zygomatic bone, (t) temporal bone, (e) ear; EMG transducer: (m) masseter muscle. The right picture shows the graphical user interface (GUI), push button (b), and two data acquisition units (DAQs).

The experimental setup consisted in total of eight bone‐conducting transducers: six generic bone‐conduction transducers (MEAS, Dortmund, Germany) and two voice‐pick‐up bone transducers (Sonion, Hoofddorp, The Netherlands), as illustrated in Figure 1. The voice‐pick‐up transducers were integrated into two commercial earpieces that occluded the ear (this type of ear closure will be referred to as “semioccluded” in this paper). Using medical tape, the remaining six transducers were attached to the participant's head at the frontal, zygomatic, and temporal bones. The participant was given a push button to press during active periods of jaw clenching, teeth grinding, reading, eating, and drinking to label data from these activities.

Also, three EMG electrodes were attached to each side of the head (Advancer Technologies, USA) to monitor the activity of the masseter muscles.

The data from them were not used for this article. The transducers and the push button were directly connected to two data‐acquisition devices (DAQs; MCC, Bietigheim‐Bissingen, Germany). Verbal and nonverbal sounds recorded via bone and tissue conduction have a limited bandwidth of less than 2 kHz (Prinz 2000; Ashbrook et al. 2016; Bouserhal et al. 2018; Chabot et al. 2021). For this study, the highest informative frequency was set at 3 kHz, leading to a sampling rate of 6 kHz. The data acquisition devices were connected to a PC via USB to store the data. Lastly, a graphical user interface realized with Unity, a game development platform (Unity Technologies, California, USA), was used to provide the participant with the cues and timers associated with the tasks. This graphical user interface was developed in‐house to guide the participant. It consisted of multiple slides notifying the participant of their task. Also, a timer was displayed on the screen to inform the participant of the time required to perform a certain task.

### 2.2 Participants

Fifteen volunteers (seven males and eight females, aged 24–40 years, median age: 31 years) participated in this study. They were recruited in Basel, Switzerland. The investigation was conducted after receiving approval by the regional ethics committee (Ethikkommission Nordwest‐ und Zentralschweiz, Application Number: 2021‐002266). Each participant signed an informed consent form.

The inclusion criteria were the ability to speak, read, and write in English or German, an age between 18 and 50 years, and the provision of a signed consent form. The exclusion criteria were dental implants (removable full or partial dentures), orofacial pain, facial beard piercing, pregnancy, not being able to complete the required tasks due to language or psychological obstacles, allergy to silicon or medical tape, ear problems, wearing a hearing aid, COVID‐19 symptoms, and involvement in the study design or family members, staff, or individuals dependent on people involved in the study design.

### 2.3 Experimental Protocol

First, the information about the study was discussed with the participant to prevent misunderstandings. Then, the participant was asked to fill out a questionnaire to collect general information on the participant's oral‐health status, as we wanted to exclude participants with an oral‐health status where voluntary oral behaviors could lead to damage. The transducers were then attached to the participant's head, and a condensed version of the main experiment was conducted to familiarize the participant with the setup. The experiment was divided into six tasks: T_1 (jaw clenching), T_2 (teeth grinding), T_3 (jaw clenching), T_4 (reading), T_5 (eating), and T_6 (drinking); between each task, the participant was allowed a 1‐min break.

**Figure 2:**
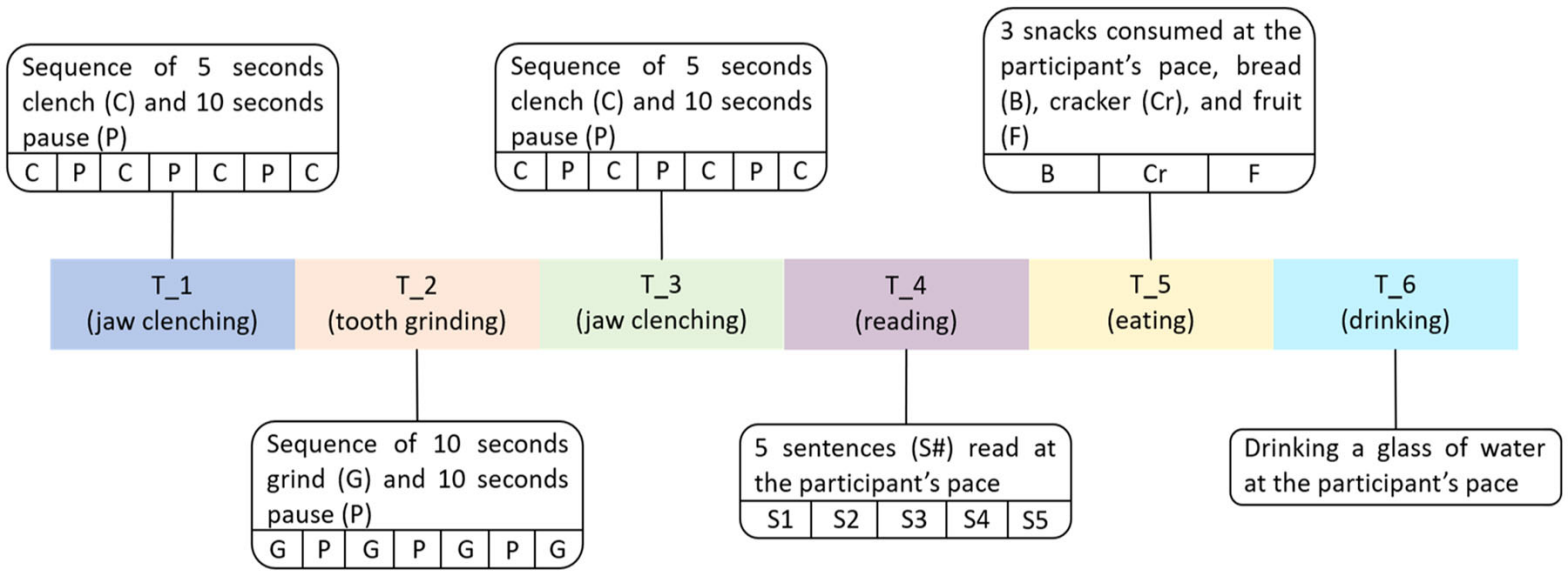
Experimental tasks: T_1 (jaw clenching), T_2 (teeth grinding), T_3 (jaw clenching), T_4 (reading), T_5 (eating), and T_6 (drinking).

Each participant was asked to sit in front of a computer screen that served as a guide throughout the experiment. The participant was asked to press the push button when performing an activity such as clenching, grinding, reading, eating, and drinking. As illustrated in Figure 2, each task was divided into different periods; for instance, T_1 and T_3 were sequences of jaw clenching and pausing periods, and T_2 was a sequence of teeth grinding and pause periods. The participants were asked to grind their teeth with as much force as they can without hurting themselves. During T_4, the participant read the fable “The North Wind and The Sun,” divided into five sentences (International Phonetic Association 1999). During T_5, the participant was asked to eat three different snacks: a piece of bread, a cracker, and a fruit. In T_6, the participant was asked to drink at least three sips of water. Finally, the participant was asked to sit quietly for 1 min to record a pause period. At the end of the experiment, the participant was asked to fill out a second questionnaire to evaluate their experience; the only goal of this was to improve our setup and protocol for future experiments. These answers to the first questionnaire were only used to check if the inclusion criteria were satisfied, so the answers are not reported. The two questionnaires are available in Files S2 and S3. The evaluation of jaw‐clenching behavior was divided into two tasks (T_1 and T_3) to avoid any unnecessary loads on the participant's joints. In total, the six tasks resulted in recording the five oral behaviors: jaw clenching, teeth grinding, reading, eating, and drinking. The study information and the questionnaires are provided as Supporting Information.

### 2.4 Data Processing

The data obtained from the study were processed with Matlab 2019b (Mathworks, Massachusetts, USA). Recorded data for each transducer were filtered with a least‐squares linear‐phase FIR low‐pass filter (1000 Hz cut‐off frequency, order of 20, and 30% transition window) and a least‐squares linear‐phase FIR high‐pass filter (50 Hz cut‐off frequency, order of 15, and a 20% transition window). Spectral subtraction was applied to the filtered data following the work of Zavarehei (2021). First, we segmented the recording into 1‐s windows and calculated the energy for each window using Equation (1). The window with the lowest energy was assumed to contain any remaining noise. Then, the completely filtered recording and the 1‐s window with the lowest energy were converted to the spectral domain. Afterward, the power and the phase spectrums obtained for the 1‐s window with the lowest energy were removed from the complete filtered recording. Finally, the output of the spectral subtraction was reconstructed for the time domain from the spectral domain.

Afterward, the processed data were segmented into overlapping windows of 100 ms length and a 50% overlap with the next window to obtain the following metrics. Three metrics were investigated: energy level in the time domain, flux in the spectral domain (Giannakopoulos and Pikrakis 2014), and zero‐crossing rate (ZCR). The energy level reflects the change between high‐energy and low‐energy periods, such as reading and drinking. The spectral flux allows the estimation of the change in spectral power between two consecutive windows. In addition, ZCR helps distinguish between active periods and inactive periods because the lower the rate, the higher the likelihood that the window contains valuable information (Giannakopoulos and Pikrakis 2014).

The energy level was estimated using the following equation:

(1) $$E^{w}=\frac{1}{N_{w}}\;\sum_{i=1}^{N_{w}}{\left|x_{i}\right|}^{2},$$

where *E*_w_ is the average energy of a window, *N*_w_ is the window's size, and *x*_*i*_ is the data point at time step *i* obtained from the processed data.

The spectral flux, which reflects the change in spectrum between successive windows, was obtained using Matlab's 2019b built‐in function spectralFlux.

ZCR was obtained for each transducer and for each of the 15 participants using the following equation:

(2) $${\mathrm{ZCR}}_{w}=\frac{1}{N_{w}}\sum_{i=1}^{N_{w}-1}f(x_{i+1}*x_{i}),\;\mathrm{with}\;f\left(\lambda \right)=\left\{\begin{array}{l} 1,\;\mathrm{if}\;\left(\lambda \right)<0,\\ 0,\;\mathrm{otherwise}, \end{array}\right.$$

where *ZCR*_w_ is the window's ZCR, *N*_w_ is the window's size, *x*_*i* + 1_ is the post‐processed data point at time step *i* + 1, and *x*_*i*_ is the post‐processed data point at time step *i* obtained from the processed data. The data point represents the post‐processed transducer's output at each time step.

For each participant and for each transducer output, the periods when the push button was pressed and the pause period at the end were segmented from the full recording after the data were processed. Then, the periods were divided into 100 ms windows with 50% overlap to obtain the energy, flux, and ZCR; the mean value of the total windows per task for each participant and transducer was used to perform the statistical analysis. For statistical analysis, examining the significant difference between the various transducer placements on energy, flux, and ZCR, a two‐way ANOVA was used with a significance level of alpha 0.05. A pairwise comparison *t*‐test between the different locations was performed using the Bonferroni correction. For both examinations, the two‐way ANOVA and the pairwise tests, Matlab 2019b built‐in functions were used: ANOVA2 and Multcompare, respectively. In addition, for some transducers, an additional nonparametric test was conducted using Matlab's rank‐sum function. This step was done because from some distributions, the normality assumption was strongly violated. We have included in the Supporting Information the data (File S4: Data.xlsx) that were used for the statistical tests and a script to produce the Q–Q plots for the transducers that exhibited a significant difference using ANOVA2 and the pairwise test, and to run the statistical tests.

## 3 Results

**Figure 3:**
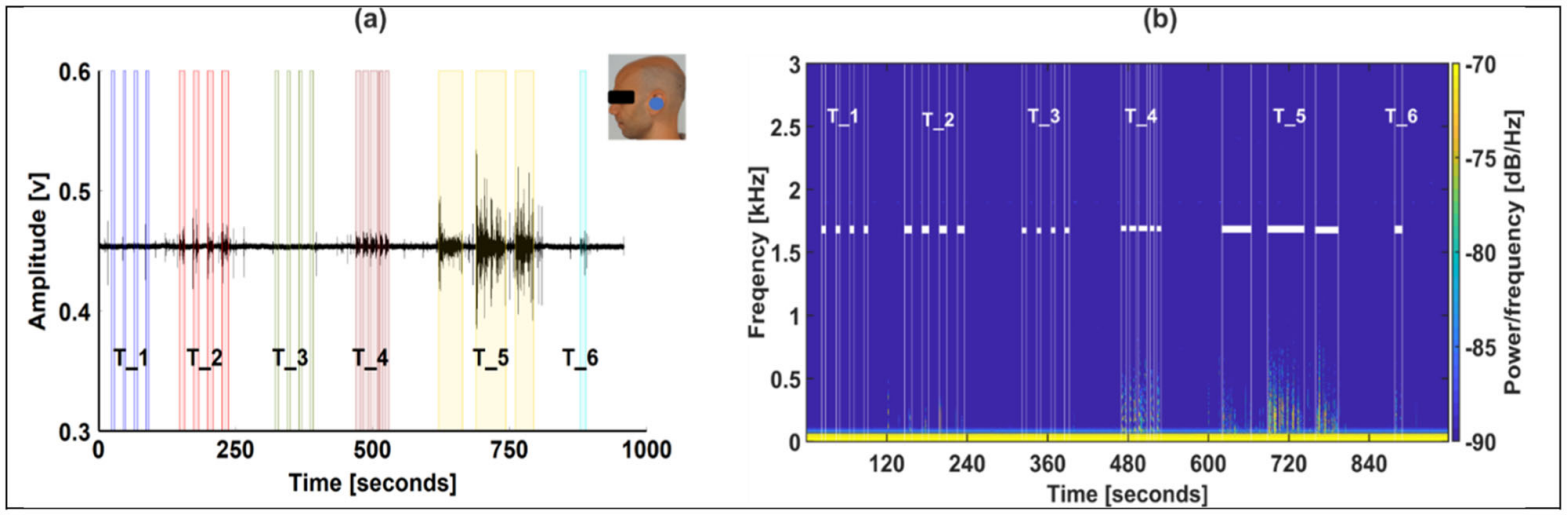
Plot of the time and frequency domains of the left‐ear transducer for Participant 3 before processing. The shaded areas in (a) represent the periods when the participant was active as recorded by push‐button input. The active periods were represented in the frequency domain plot in (b) as the area between the white lines. The experimental tasks were as follows: T_1: jaw clenching; T_2: teeth grinding; T_3: jaw clenching; T_4: reading; T_5: eating; T_6: drinking.

The output of the left‐ear transducer in the time and frequency domains before processing for Participant 3 is illustrated in Figure 3a,b. T_5 (eating) has the largest peak‐to‐peak range of [0.38 0.53] compared to [0.41 0.48], [0.43 0.46], and [0.42 0.46] for T_2 (teeth grinding), T_4 (reading), and T_6 (drinking), respectively, whereas the clenching tasks, T_1 (jaw clenching) and T_3 (jaw clenching), were not observed, as illustrated in Figure 3a. Supporting Information S1: Figure 9a,b illustrates the output of the transducer after processing the data as described in Section 2. In Supporting Information S1: Figure 9a, the signal range was reduced by a factor of 10. Similarly, T_5 had the largest peak‐to‐peak range of [−0.06 0.08], compared to [−0.03 0.02], [−0.01 0.01], and [−0.02 0.01] for T_2, T_4, and T_6, respectively. The clenching tasks, T_1 and T_3, did not result in a change in audio recording in the time domain. As illustrated in Supporting Information S1: Figure 9b, T_2, T_4, T_5, and T_6 were below 1 kHz.

**Figure 4:**
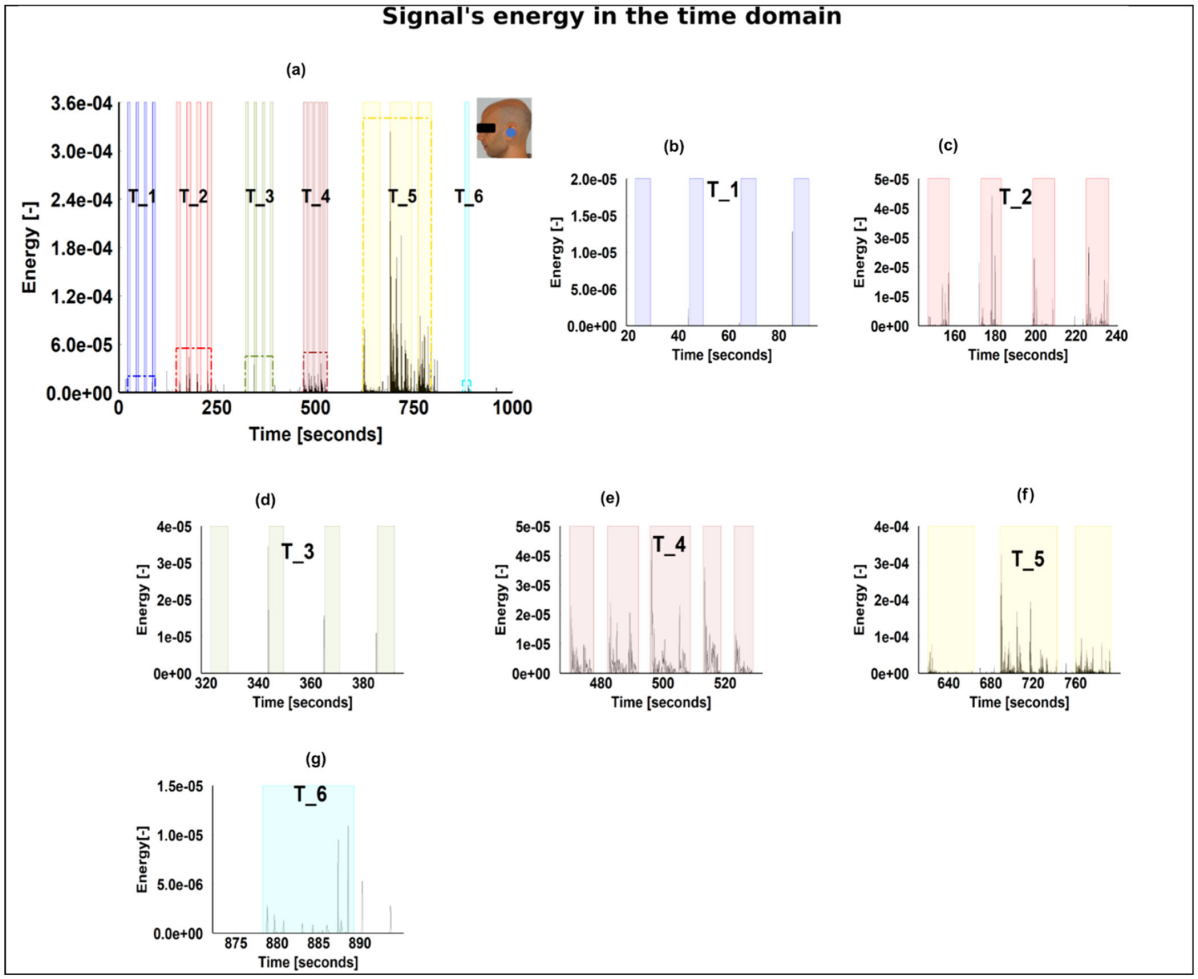
The energy of the signal in the time domain. (a) Energy obtained from the left ear of one participant (in this case Participant 3); (b)–(g) magnification of the various tasks illustrated in (a). The experimental tasks were as follows: T_1: jaw clenching; T_2: teeth grinding; T_3: jaw clenching; T_4: reading; T_5: eating; T_6: drinking.

**Figure 5:**
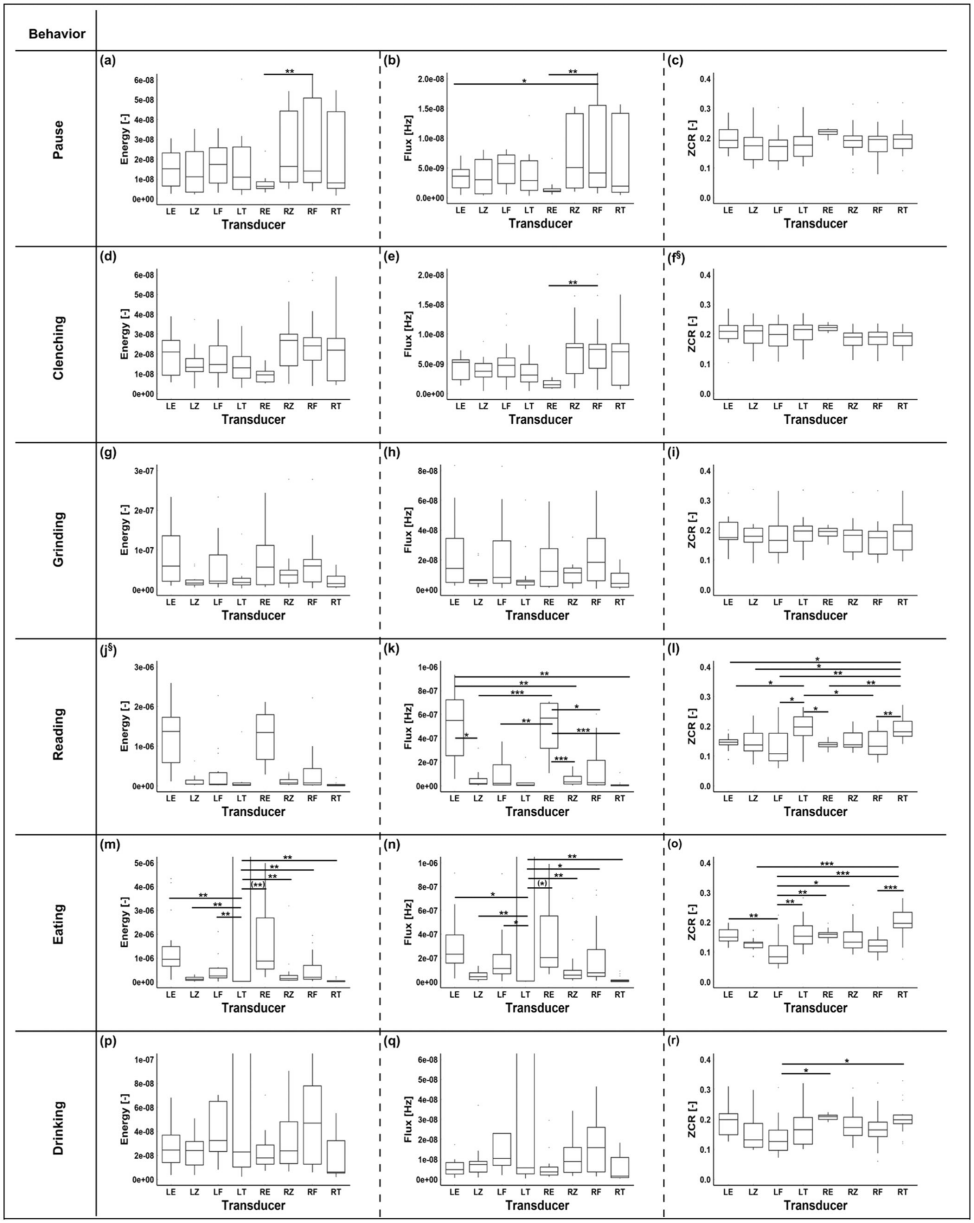
Energy, flux, and ZCR of all participants for each transducer and each behavior: clenching (a–c), grinding (d–f), reading (g–i), eating (j–l), drinking (m–o), and pause (p–r). The transducers are depicted on the horizontal axis as follows: left ear (LE), left zygomatic (LZ), left frontal (LF), left temporal (LT), right ear (RE), right zygomatic (RZ), right frontal (RF), and right temporal (RT). The marks inside the boxes represent the median, and the bottom and top of the boxes represent the 25th (q1) and the 75th (q3) percentiles, respectively. Significant differences between the different transducer placements are indicated with * for *p* < 0.05, ** for *p* < 0.01, and *** for *p* < 0.001. A full‐range illustration of figures (j), (k), (m), and (n) can be found in Supporting Information S1: Figure 1. §: Showed a significant difference as listed in Table 1 but was not included in the pairwise tests. The *p* values for the pairwise tests are listed in Supporting Information S1: Table 2. Note: (**) and (*) were added to two subfigures (m and n); they refer to instances where an additional nonparametric test was performed and the result was opposite to that of the ANOVA.

The spectrograms obtained from the rest of the transducers are illustrated in Supporting Information S1: Figures 2–8.

Figure 4a–g illustrates the signal's energy in the time domain obtained using Equation (1) for Participant 3's left‐ear transducer with a magnified view of the six tasks. The shaded areas represent the active periods where the participant was asked to intentionally perform one of the tasks listed in Figure 2 while pressing the push‐button input. The peak amplitude for tasks T_2, T_4, T_5, and T_6 are listed in Supporting Information S1: Table 1. T_5 had the highest energy (arbitrary unit), which is seven times higher than tasks T_2 and T_4 and 30 times higher than T_6. Figure 4b,d shows that the clenching tasks had a relatively negligible amount of energy within the shaded areas. However, a peak can be seen just before some of the shaded areas in both figures. Supporting Information S1: Figure 10a–h illustrates the energy of the signal for T_2 obtained from the eight transducer placements for Participant 3. As illustrated in Supporting Information S1: Figure 10a–d, the left‐ear transducer had the highest energy, which was approximately eight times higher than that of the right‐ear transducer and 30% higher than that of the right‐temporal transducer, which is illustrated in Supporting Information S1: Figure 10e and 10h, respectively. The recording amplitude of the remaining transducers was almost negligible, as illustrated in Supporting Information S1: Figure 10b–d,f,g.

Different transducer placements were analyzed for each behavior, and the *p* values—which were obtained for each metric using the mean values of the behavior periods of each participant—are displayed in Table 1. A significant effect can be found for the pause period when examining energy and flux. For the clenching behavior, a significant difference can be found among the different transducer placements for flux and ZCR. Regardless of the metric used, the placement of the transducer did not have a significant effect when teeth‐grinding behavior was being examined. The placement of the transducer did, however, significantly impact the recording quality for both reading and eating, regardless of the metric under investigation. Finally, only when the ZCR output of drinking was being investigated did the transducer placement show a significant influence.

**Table 1:** The *p* value of a two‐way ANOVA test for the different behaviors using energy, spectral flux, and ZCR.

Metric	Behavior					
	Clenching	Grinding	Reading	Eating	Drinking	Pause
Energy	*F*(7) = 1.15	*F*(7) = 1.34	***F*** **(7)** = **2.5**	***F*** **(7)** = **3.6**	*F*(7) = 1.42	***F*** **(7)** = **2.55**
	*p* > 0.05	*p* > 0.05	***p*** < **0.05**	***p*** < **0.01**	*p* > 0.05	***p*** < **0.05**
Flux	***F*** **(7)** = **3.06**	*F*(7) = 0.85	***F*** **(7)** = **6.77**	***F*** **(7)** = **3.39**	*F*(7) = 1.28	***F*** **(7)** = **3.16**
	***p*** < **0.01**	*p* > 0.05	***p*** < **0.001**	***p*** < **0.01**	*p* > 0.05	***p*** < **0.01**
ZCR	***F*** **(7)** = **2.39**	*F*(7) = 0.48	***F*** **(7)** = **5.24**	***F*** **(7)** = **8.4**	***F*** **(7)** = **3.34**	*F*(7) = 1.4
	***p*** < **0.05**	*p* > 0.05	***p*** < **0.001**	***p*** < **0.001**	***p*** < **0.01**	*p* > 0.05

*Note*: *F* is the *F*‐statistic and the value 7 represents the degrees of freedom (the different transducer placements). Bold values indicate statistically significant at *p* < 0.05.

Figure 5 presents the box plots of every behavior for each of the three metrics, energy, flux, and ZCR, obtained from the mean values of each behavior for the 15 participants. Superimposed on top of the box plots are the results of the pairwise tests for each behavior and each metric that already yielded a significant difference (*p* < 0.05), as shown in Table 1. The *p* values of each pairwise test are listed in Supporting Information S1: Table 2. Notably, reading and eating had the highest medians for energy and flux. In addition, the placement of the transducer had a significant influence on spectral flux and ZCR for both eating and reading as illustrated in Figure 5g–l. Also, the transducer's location affected the energy of the eating task and the pause period.

## 4 Discussion

We recorded sounds of teeth grinding, reading, eating, and drinking in comparison to the pause period as illustrated in Figure 5. We noticed that the location of the transducer particularly impacted the amplitudes of the metrics used for the behaviors of reading and eating. However, for teeth grinding, the location of the transducer did not have any significant impact, as illustrated in Figure 5.

By examining the representation of the pause period illustrated in Figure 5a–c, one can see that the placement of the transducer had no significant impact on ZCR, and the differences between the placements could be related to variations in the background noise. However, for energy and flux, the sensitivity was a bit higher, and some placements significantly impacted the output listed in Supporting Information S1: Table 2. That could also be related to background noise variation or the displacement of certain transducers.

The ranges of energy, flux, and ZCR for clenching and the pause period did not differ significantly, as illustrated in Figure 5a–f. This observation can be attributed to the occlusion type and the transducer placement in the earpiece. Such inference is supported by a pilot study comparing a fully occluded ear with a semioccluded ear (Nahhas et al. 2022). It indicated that the type of occlusion and the placement of the transducer in the ear canal affect the level of isolation the ear canal is enduring to record such a relatively weak signal. Nonetheless, for this particular study, the semiocclusion approach was used for hygienic and practical reasons. The clenching behavior showed a distinct feature: a peak just before the shaded areas, as illustrated in Figure 4b–d. This peak could be related to tooth–tooth contact, as the participant was preparing to clench. This interpretation of the peak is supported by the fact that its amplitude is similar to that of the grinding behavior as illustrated in Figure 4c. This could mean that either this peak is a characteristic of the behavior itself or is due to the protocol that requires the participant to perform certain actions intentionally, which could alter the behavior itself. This requires further investigation.

For teeth grinding, the energy and flux ranges were twofold higher than the pause period. This observation reflects the possibility of recording teeth‐grinding sounds from different locations on the head. The placement of the transducer had no significant impact on the energy and flux, as illustrated in Figure 5g,h and listed in Table 1. The ear transducers had the highest 75th percentile, as illustrated in Figure 5g, whereas for flux, multiple locations, including the ear, were advantageous to recording teeth grinding, as reflected in the relatively high 75th percentile, but the location of the transducer did not have any significant impact, as illustrated in Figure 5h and listed in Table 1. In addition, the placement of the transducer did not have a significant impact on ZCR, as illustrated in Figure 5i, which suggests that the signal‐to‐noise ratio might be relatively constant when such behavior is being recorded, as ZCR mirrors the noisiness of the signal.

For reading, energy and flux were twofold higher than that of the pause period, as illustrated in Figure 5j,k. Both energy and flux reflected that the ear transducers had the highest median and 75th percentile; this might be related to the properties of the transducers that are tuned to record voice. Another factor could be the occlusion effect. Occlusion of the ear increases the strength of the bone‐conducted signal, as noted by Bouserhal et al. (2018). Noting that the type of occlusion used in this study is a semiocclusion. Both flux and ZCR were sensitive to the placement of the transducer, as illustrated in Figure 5k,l and listed in Supporting Information S1: Table 2. However, energy was less sensitive to reflect the significant impact of the placement inferred by the ANOVA test listed in Table 1.

For eating, the three metrics energy, flux, and ZCR, reflected that the placement of the transducer significantly impacted the recording, as listed in Table 1. The left‐temporal transducer had the highest median and 75th percentile for energy and flux. Such a stark difference can be attributed to the transducer adjustment after it was detached. The likelihood for such an explanation is supported by the experiment's timetable illustrated in Figure 2, since a similar observation can be noticed for drinking, which succeeded eating. However, such an observation is absent in the preceding behavior, grinding and reading, as illustrated in Figure 5h and 5k, respectively.

For both reading and eating, the impact of the transducer placement on the recording can be attributed to the behavior itself, since the recording became weaker or varied with time. While chewing, the signal‐to‐noise ratio changed with time, since the consistency and properties of the consumed snacks changed. Additionally, other factors contributed to this variation, such as differences in the distance between the location of the transducer and the source of the sound and the different tissues that the signal had to pass through. For reading, similar observations were made to eating, since different words had different characteristics that affected the signal. In addition, the speed and the loudness of the act of reading or eating affected the signal‐to‐noise ratio, and ZCR reflected the noisiness of the signal (Giannakopoulos and Pikrakis 2014).

For drinking, the placement of the transducer had a significant impact on ZCR as listed in Table 1. Nonetheless, the output of the transducers had a relatively prominent difference, as illustrated in Figure 5l,m. The difference can be related to the transducer displacement caused by the behavior, as the participants might have tilted their heads. On the other hand, the noise level was significantly different between some of the placements, as ZCR demonstrated a relatively high sensitivity compared to energy and flux as listed in Supporting Information S1: Table 2.

## 5 Limitations

The transducers' attachment differed for each participant due to anatomy, such as the size of their head, their skin properties, and the size of their masseter muscles. In addition, the standard earpiece used in this study did not completely occlude the ear due to the participants' different ear sizes and the properties of the rubber tip of the earpiece. Also, the position of the transducer in the earpiece differed slightly between the left and the right sides. The volunteers were not medically assessed for bruxism, and the tasks associated with bruxism‐like events were done on a voluntary basis. Thus, the movements did not accurately represent the behavior under investigation. The participants might have relied more on one side of the jaw while grinding and eating, resulting in an imbalanced distribution of sounds.

## 6 Conclusion

Based on the results of this study, we can conclude that the acoustic emission of various oral behaviors can be recorded from the head. However, we were not able to record characteristic features of jaw clenching except for a peak observed just before the probable onset. From the observed differences, we can conclude that the position of the transducer affected the quality of the recording. Although the placement of the transducer did not significantly impact the recording of teeth‐grinding sounds, the ear is a good location for transducer placement compared to other locations, since the ear compensates for variances generated by behaviors such as eating and reading or physical requirements such as drinking. Wearing an earplug for an extended period may therefore be a trade‐off for recording such sounds during everyday activities.

## Author Contributions

Mohammad Khair Nahhas, Nicolas Gerig, and Georg Rauter contributed to the conceptualization of this work. All the authors made substantial intellectual contributions to refining, finalizing, and approving the manuscript for publication.

## Conflicts of Interest

The authors declare no conflicts of interest.

## Supporting information

Supporting information.

Supporting information.

Supporting information.

Supporting information.

## Data Availability

The data that support the findings of this study are available upon reasonable request from the corresponding author.
