# Supplementary material for: Toward Wearables for Bruxism Detection: Voluntary Oral Behaviors Sound Recorded Across the Head Depend on Transducer Placement
Source: Clin Exp Dent Res. 2024 Sep 22;10(5):e70001. doi: 10.1002/cre2.70001 (PMC11417139; doi:10.1002/cre2.70001)
Supplement: Supplementary file 1 — Supporting information. [file CRE2-10-e70001-s001.docx]

1

2 Supplementary material

3


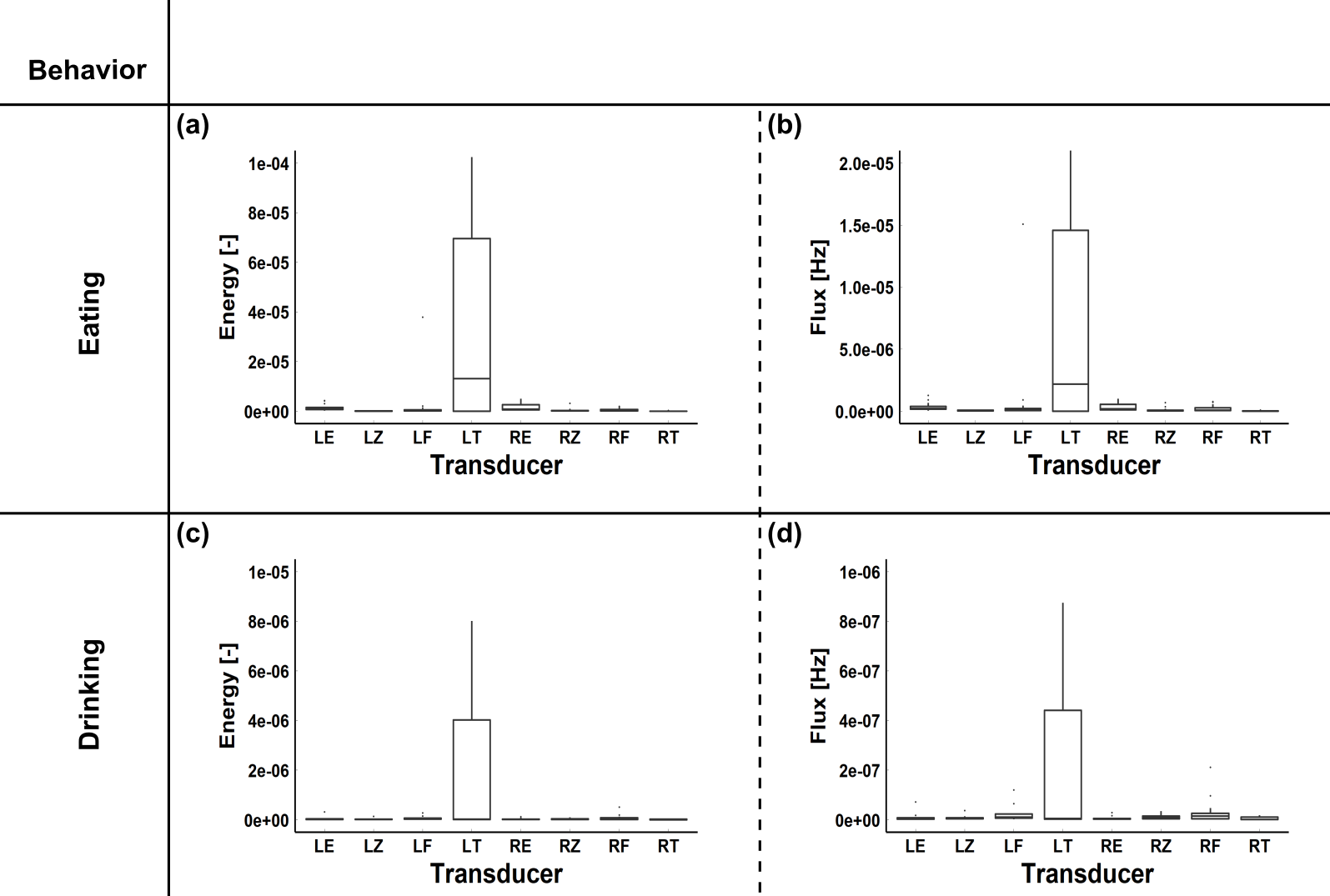


Figure 1: Energy and flux of all participants obtained for each transducer for: eating (a and b) and drinking (c and d). The marks in each box represent the median value and the bottom and top of the boxes represent the 25th (q1) and the 75th (q3) percentiles, respectively.

Table 1: Peak energy and spectral flux levels for each task within the shaded areas for third participant as determined by the left ear transducer.

| **metric** | **Tasks** | | | | | |
| --- | --- | --- | --- | --- | --- | --- |
|  | **T 1** | **T 2** | **T 3** | **T 4** | **T 5** | **T 6** |
| **Energy** | - | 4.4e-5 | - | 4.57e-5 | 32e-5 | 1.09e-5 |
| **Flux** | - | 1.07e-5 | - | 1.88e-5 | 6.58e-5 | 2.26e-6 |

4

2

Table 2: The *p*-value for the post hoc test with Bonferroni correction. The placement of the transducers is denoted by the initial letter of the side of the head (L: Left or R: Right) and the initial letter of the anatomical location (E: Ear, Z: Zygomatic, F: Frontal, and T: Temporal). LE: Left Ear, LZ: Left Zygomatic, LF: Left Frontal, LT: Left Temporal, RE: Right Ear, RZ: Right Zygomatic, RF: Right Frontal, and RT: Right Temporal.

| **Metric** | **Behavior** | | | | | |
| --- | --- | --- | --- | --- | --- | --- |
|  | **Clenching** | **Grinding** | **Reading** | **Eating** | **Drinking** | **Pause** |
| **Energy** | - | - | - | *p*(LE-LT) = 0.007  *p*(LZ-LT) = 0.006  *p*(LF-LT) = 0.008  *p*(LT-RE) = 0.007  *p*(LT-RZ) = 0.006  *p*(LT-RF) = 0.006  *p*(LT-RT) = 0.006 | - | *p*(RE-RF) = 0.007 |
| **Flux** | *p*(RE-RF) = 0.002 | - | *p*(LE-LZ) = 0.01  *p*(LE-RZ) = 0.009  *p*(LE-RT) = 0.003  *p*(LZ-RE) = 0.0002  *p*(LF-RE) = 0.005  *p*(RE-RZ) = 0.0002  *p*(RE-RF) = 0.01  *p*(RE-RT) = 0.0001 | *p*(LE-LT) = 0.01  *p*(LZ-LT) = 0.009  *p*(LF-LT) = 0.015  *p*(LT-RE) = 0.01  *p*(LT-RZ) = 0.009  *p*(LT-RF) = 0.01  *p*(LT-RT) = 0.009 | - | *p*(RE-RF) = 0.002  *p*(LE-RF) = 0.03 |
| **ZCR** | - | - | *p*(LE-LT) = 0.02  *p*(LE-RT) = 0.01  *p*(LZ-RT) = 0.03  *p*(LF-LT) = 0.01  *p*(LF-RT) = 0.006  *p*(LT-RE) = 0.01  *p*(LT-RF) = 0.01  *p*(RE-RT) = 0.005  *p*(RF-RT) = 0.008 | *p*(LE-LF) = 0.005  *p*(LZ-RT) = 0.0001  *p*(LF-LT) = 0.001  *p*(LF-RE) = 0.003  *p*(LF-RZ) = 0.01  *p*(LF-RT) = 3e-8  *p*(RF-RT) = 0.0001 | *p*(LF-RE) = 0.01  *p*(LF-RT) = 0.01 | - |

| 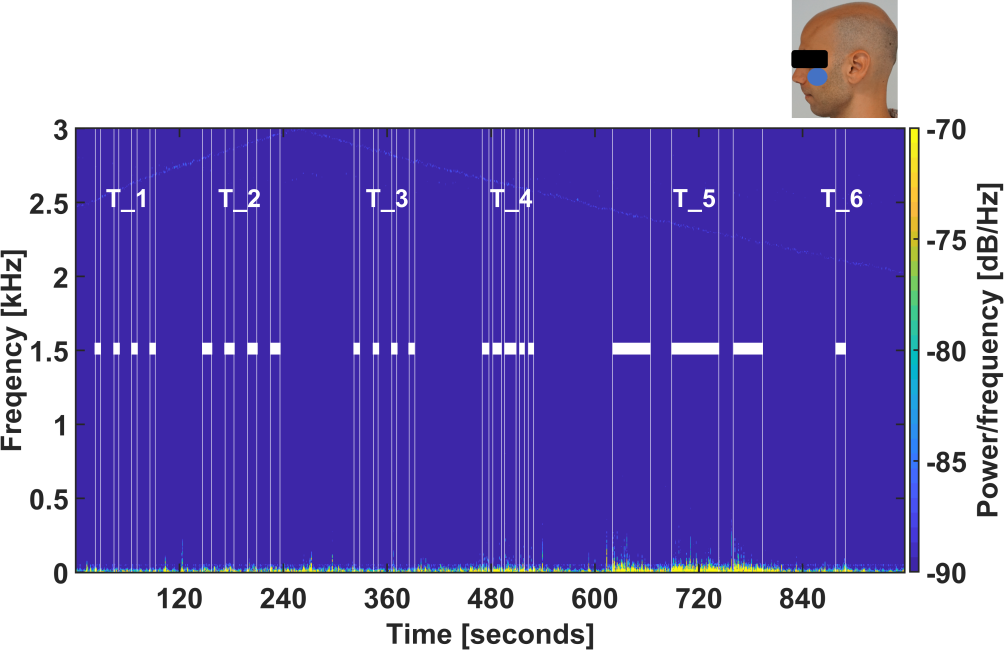 |
| --- |
| Figure 2: Spectrogram for the unprocessed recording from the left zygomatic transducer. |

| 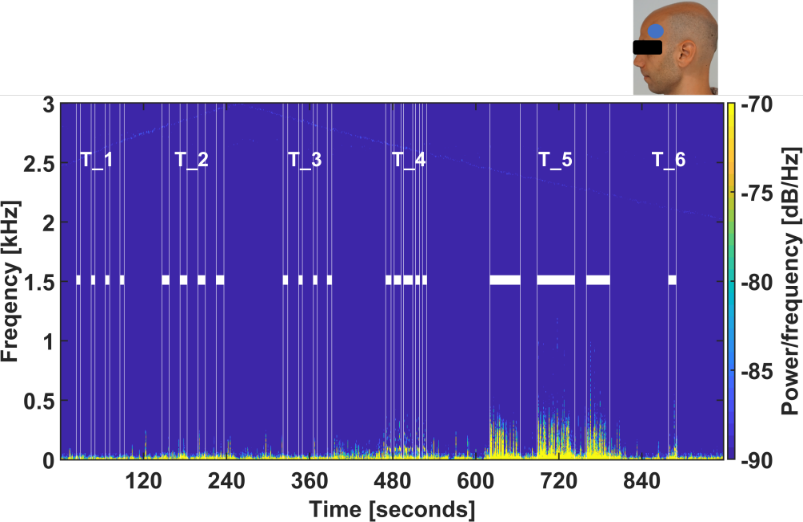 |
| --- |
| Figure 3: Spectrogram for the unprocessed recording from the left frontal transducer. |

| 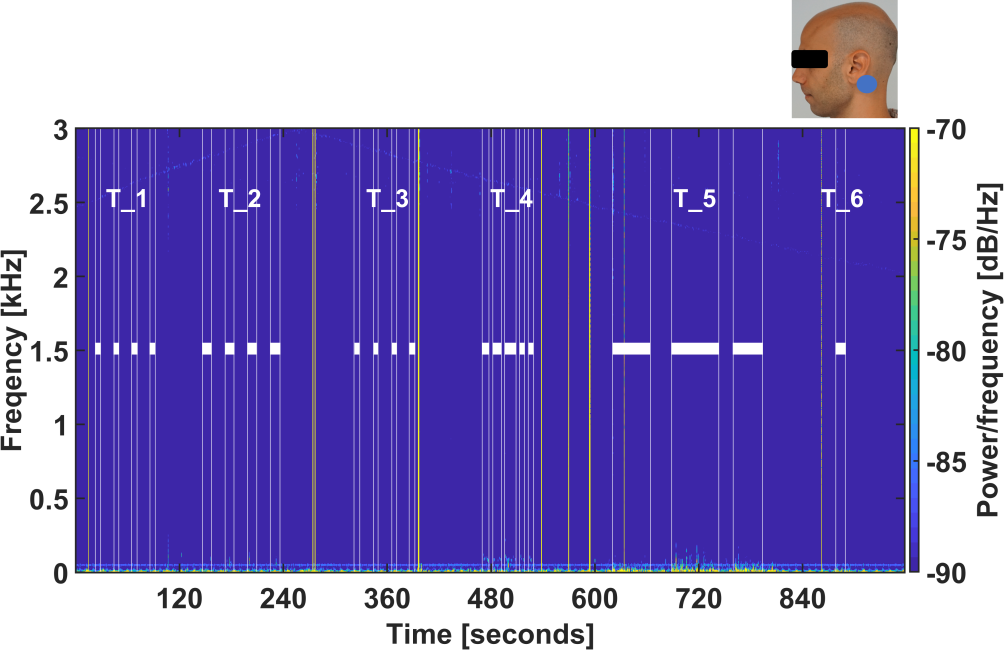 |
| --- |
| Figure 4: Spectrogram for the unprocessed recording from the left temporal transducer. |

| 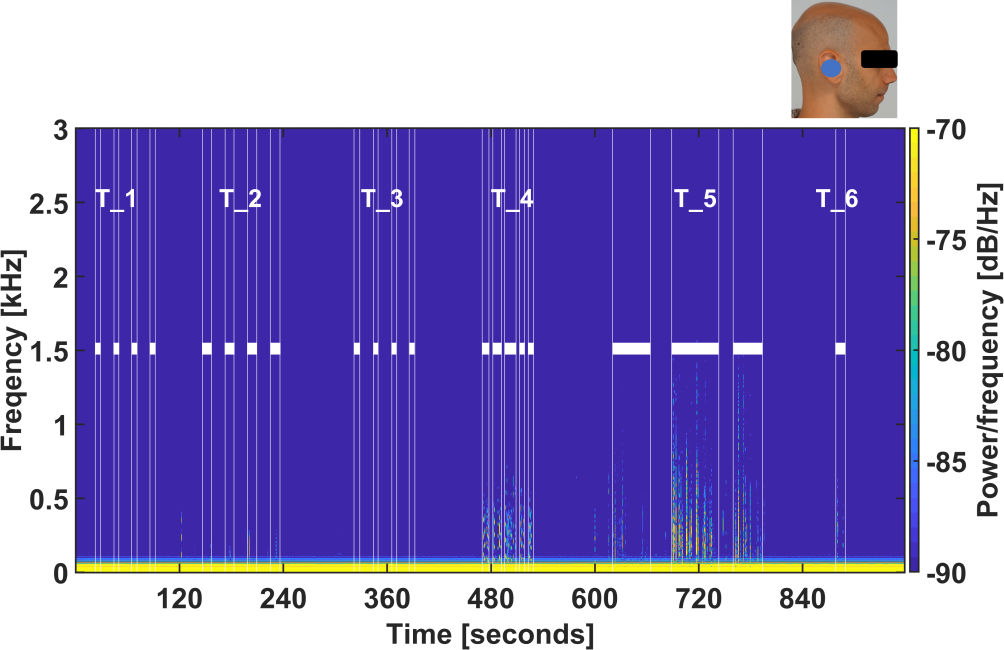 |
| --- |
| Figure 5: Spectrogram for the unprocessed recording from the right ear transducer. |

| 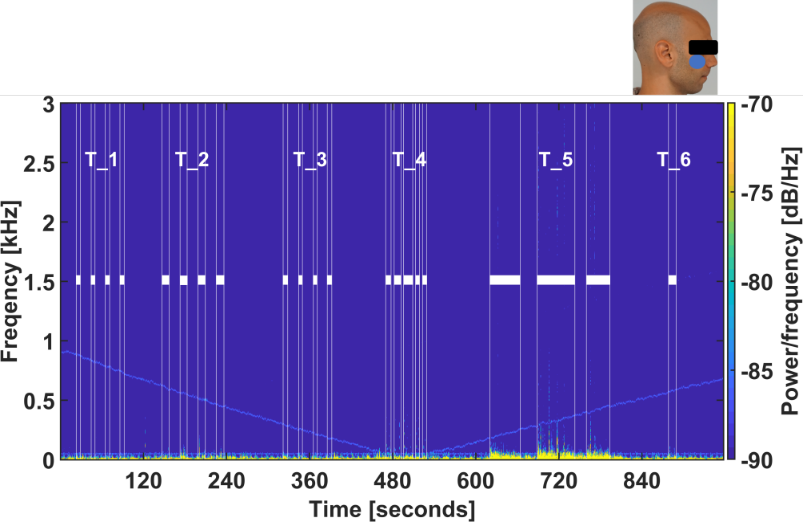 |
| --- |
| Figure 6: Spectrogram for the unprocessed recording from the right zygomatic transducer. |

| 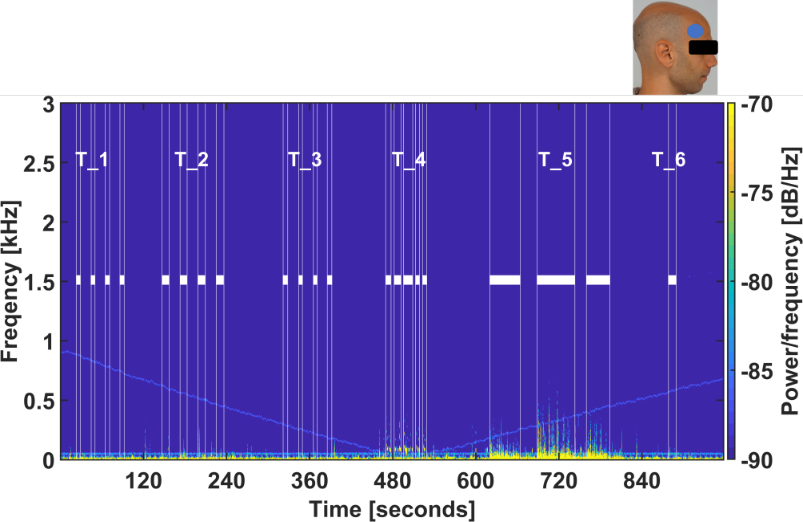 |
| --- |
| Figure 7: Spectrogram for the unprocessed recording from the right frontal transducer. |

| 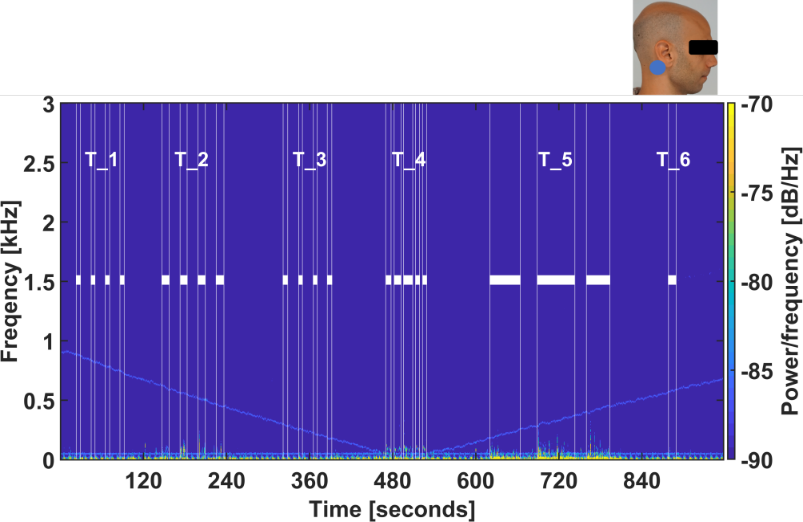 |
| --- |
| Figure 8: Spectrogram for the unprocessed recording from the right temporal transducer. |

| 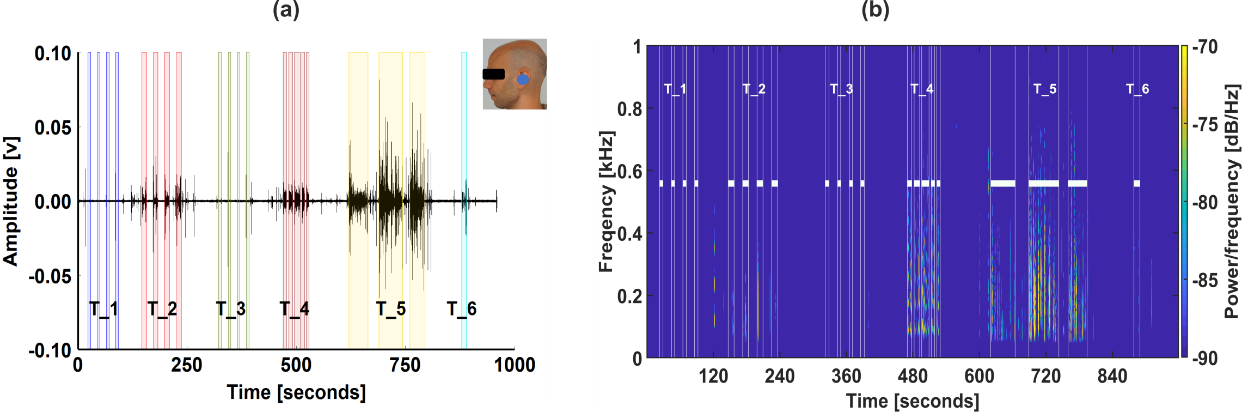 |
| --- |
| Figure 9: Plot of the time and frequency domains of the left ear transducer for participant number three after processing. The shaded areas in (a) represent the periods during which the participant was active as recorded by push button input. The active periods were represented in the frequency domain plots in Figure (b) as the area between the white lines. The experimental tasks were as follows: T_1: jaw clenching, T_2: tooth grinding, T_3: jaw clenching, T_4: reading, T_5: eating, and T_6: drinking. |

| 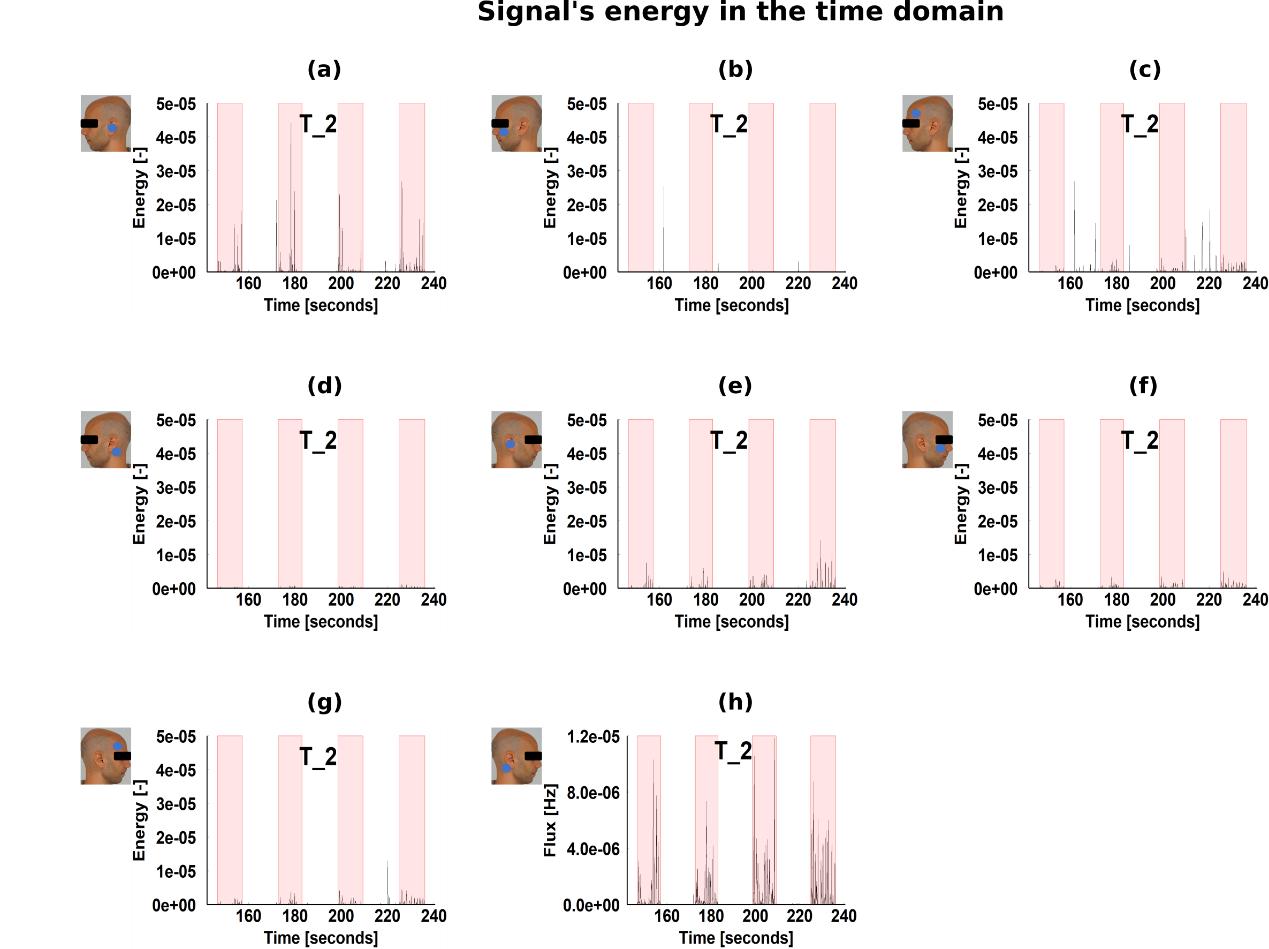 |
| --- |
| Figure 10: The energy of the signal in the time domain. Subfigures a – h represent the energy for T_2 (tooth grinding) obtained from the eight transducers for participant number three. |
