# Supplementary material for: Toward Wearables for Bruxism Detection: Voluntary Oral Behaviors Sound Recorded Across the Head Depend on Transducer Placement
Source: Clin Exp Dent Res. 2024 Sep 22;10(5):e70001. doi: 10.1002/cre2.70001 (PMC11417139; doi:10.1002/cre2.70001)
Supplement: Supplementary file 2 — Supporting information. [file CRE2-10-e70001-s004.pdf]

Questionnaire number 2: Study part "A".

Participant number: Date:

***: grade is a number that ranges from 0 till 10; whereas, 0 stands for strongly disagree and 10 stands for strongly agree.**

****: grade is a number that ranges from 0 till 10; whereas, 0 stands for no pain and 10 stands for severe pain.**

Question	Answer	
1) My tasks were clear to me:	Grade*:	
2) The user interface was easy to follow:	Grade*:	
3) Any tooth pain due to the experiment:	Yes: ☐<br>If yes, grade**:	No: ☐
4) Any masseter muscle pain due to the experiment:	Yes: ☐<br>If yes, grade**:	No: ☐
5) Any jaw joint paint due to the experiment:	Yes: ☐<br>If yes, grade**:	No: ☐
6) Any neck pain due to the experiment:	Yes: ☐<br>If yes, grade**:	No: ☐
7) Any headache due to the experiment:	Yes: ☐<br>If yes, grade**:	No: ☐
8) Any earache due to the experiment:	Yes: ☐<br>If yes, grade**:	No: ☐
9) Comments:		
