# Supplementary material for: Toward Wearables for Bruxism Detection: Voluntary Oral Behaviors Sound Recorded Across the Head Depend on Transducer Placement
Source: Clin Exp Dent Res. 2024 Sep 22;10(5):e70001. doi: 10.1002/cre2.70001 (PMC11417139; doi:10.1002/cre2.70001)
Supplement: Supplementary file 3 — Supporting information. [file CRE2-10-e70001-s003.pdf]

Questionnaire number 1: Study part “A”.

Participant number: Date:

***: mention in the answer if it was based on a professional medical assessment (M) or due to personal report (P).**

Question	Answer	
1) Age:	____________________	
2) Gender:	____________________	
3) Height:	____________________	
4) Weight	____________________	
5) Do you have bruxism?	Yes: ☐ **(grinding or clenching)**<br>If yes, since when (months) *****:	No: ☐
6) Do you have neck pain?	Yes: ☐<br>If yes, since when (months) *****:	No: ☐
7) Do you have back pain**?**	Yes: ☐<br>If yes, since when (months) *****:	No: ☐
8) Do you have jaw joint pain?	Yes: ☐<br>If yes, since when (months) *****:	No: ☐
9) Do you have masseter muscles soreness?	Yes: ☐<br>If yes, since when (months) *****:	No: ☐
10) Do you have any tooth fillings?	Yes: ☐<br>If yes, how many (approximate number):	No: ☐
